# The association between psychosocial factors and mental health symptoms in cervical spine pain with or without radiculopathy on health outcomes: a systematic review

**DOI:** 10.1186/s12891-023-06343-8

**Published:** 2023-03-28

**Authors:** Michael Mansfield, Mick Thacker, Joseph L. Taylor, Kirsty Bannister, Nicolas Spahr, Stephanie T. Jong, Toby Smith

**Affiliations:** 1 Faculty of Medicine and Health Sciences, University of East Anglia, Norwich, NR4 7TJ UK; 2 School of Sport, Exercise and Rehabilitation Sciences, College of Life and Environmental Sciences, University of Birmingham, Edgbaston, Birmingham, B15 2TT UK; 3 School of Physiotherapy, Royal College of Surgeons Ireland, 123 St Stephen’s Green, Dublin 2, Ireland; 4 Wolfson Centre of Age Related Diseases, Institute of Psychiatry, Psychology and Neuroscience, Central Modulation of Pain, King’s College London, London, SE1 1UL UK; 5 Physiotherapy Department, Guy’s and St Thomas Hospital NHS Foundation Trust, St Thomas Hospital, Westminster Bridge Road, London, UK; 6 Warwick Medical School, University of Warwick, Coventry, UK

**Keywords:** Cervical spine pain, Neck pain, Cervical spine radiculopathy, Mental health, Psychosocial, Health outcomes, Adult

## Abstract

**Background:**

Neck pain, with or without radiculopathy, can have significant negative effects on physical and mental wellbeing. Mental health symptoms are known to worsen prognosis across a range of musculoskeletal conditions. Understanding the association between mental health symptoms and health outcomes in this population has not been established. Our aim was to systematically review the association between psychosocial factors and/or mental health symptoms on health outcomes in adults with neck pain, with or without radiculopathy.

**Methods:**

A systematic review of published and unpublished literature databases was completed. Studies reporting mental health symptoms and health outcomes in adults with neck pain with or without radiculopathy were included. Due to significant clinical heterogeneity, a narrative synthesis was completed. Each outcome was assessed using GRADE.

**Results:**

Twenty-three studies were included (N = 21,968 participants). Sixteen studies assessed neck pain only (N = 17,604 participants); seven studies assessed neck pain with radiculopathy (N = 4,364 participants). Depressive symptoms were associated with poorer health outcomes in people with neck pain and neck pain with radiculopathy. These findings were from seven low-quality studies, and an additional six studies reported no association. Low-quality evidence reported that distress and anxiety symptoms were associated with poorer health outcomes in people with neck pain and radiculopathy and very low-quality evidence showed this in people with neck pain only. Stress and higher job strain were negatively associated with poorer health outcomes measured by the presence of pain in two studies of very low quality.

**Conclusions:**

Across a small number of highly heterogenous, low quality studies mental health symptoms are negatively associated with health outcomes in people with neck pain with radiculopathy and neck pain without radiculopathy. Clinicians should continue to utilise robust clinical reasoning when assessing the complex factors impacting a person’s presentation with neck pain with or without radiculopathy.

**PROSPERO registration number:**

CRD42020169497.

**Supplementary Information:**

The online version contains supplementary material available at 10.1186/s12891-023-06343-8.

## Background

Cervical spine pain with or without radiculopathy (CSp ± R) has a significant negative impact on people’s physical and mental health. It is an enormous burden for individuals, families and societies [1, 2]. The reported incidence of cervical spine radiculopathy (CSR) is between 0.83 and 1.79 per 1000 person-years, and prevalence ranges from 1.2 to 5.8 per 1000 [3]. The one-year incidence of cervical spine pain ranges between 10% and 21% [4, 5]. The global prevalence of cervical spine pain and years lived with disability has each increased by 19% over the last 10 years [6].

The association between psychological and/or mental health symptoms and LBP is well-established with low back pain [7, 8]. It is recognised that these symptoms are negatively associated with health outcomes and quality of life [7, 8]. Psychosocial factors encompass a wide range of cognitions, emotions, behaviours and family and workplace influences [9]. Mental health symptoms or conditions are an extension of such factors. Stress, anxiety, depression and negative coping behaviours negatively impact prognosis with musculoskeletal conditions such as low back pain [10], work related neck pain [11], knee osteoarthritis [12], carpal tunnel syndrome [13] and shoulder pain [14]. Psychosocial factors and/or mental health symptoms should be considered as part of a clinical reasoning framework to positively affect health outcomes and support prognosis [15]. The extent to which these factors may impact acute or persistent CSp ± R across global locations has not yet been synthesised in a systematic review study design.

Establishing the associative factors between psychosocial factors and/or mental health symptoms and health outcomes will enhance our understanding of these complex interactions. Furthermore, it should enhance clinicians’ assessment and management plans [16, 17]. To the authors’ knowledge, no systematic review has examined this association. Consequently, we report a systematic review assessing the association between psychosocial factors and/or mental health symptoms to health outcomes in adults with CSp ± R.

## Methods

This systematic review was registered with the International Prospective Register of Systematic Reviews (PROSPERO) database (Reference: CRD42020169497). The Preferred Reporting Items for Systematic Reviews and Meta-Analyses (PRISMA) statement [18] was followed. The review protocol has been previously published [19].

### Search strategy

A systematic search of the electronic databases EMBASE, CINAHL and MEDLINE (PubMed) from inception to 31st April 2021 was completed by one reviewer (NS) under the supervision of a second (MM). The search was updated by the lead reviewer (MM) from 31st April 2021 to 1st September 2022. The PubMed search strategy is presented in Appendix 1. Unpublished (grey) literature search and trial registry was searched (e.g., WHO.It, ZETOC, British library higher education thesis deposits). All included studies underwent reference checking.

### Eligibility criteria

Studies were included if they met the following criteria:

- A sample that included adults aged 18 years and over with CSp ± R. Following the International Association of the Study of Pain [20] and The Bone and Joint Decade 2000–2010 Task Force on Neck Pain [21] cervical spine pain definitions. We defined neck pain as cervical spine pain perceived anywhere in the posterior neck region to the first thoracic spinous process. Furthermore, a pragmatic approach was undertaken, and studies with *probable* or *definite* cervical spine radiculopathy diagnoses were adapted from IASP and North American Spine Society were eligible for inclusion [20, 22, 23] (Supplementary file 1).
- Assessed psychosocial factors or mental health symptoms as an exposure. Studies must have investigated one or more psychosocial or mental health symptoms (or conditions). Psychosocial factors may have included: cognitive (e.g., neuropsychological functioning), affective (e.g., distress, mood), behavioural (e.g., coping strategies), vocational (e.g., job satisfaction, self-perceived work ability) or interpersonal processes (e.g., social support) [24]. Mental health symptoms and conditions such as depressive symptoms, clinical depression, anxiety, perceived stress, personality, psychotic, traumatic and eating disorders were also considered. Self-reported, objective, standardised questionnaires (e.g., Beck Depression Index, Karasek’s Job Control Questionnaire, GHQ-12) and psychosocial factors or mental health symptoms assessed using dichotomous data (“yes/no”) were also considered. Studies were also eligible if the study population compared different severities of mental health symptoms, conditions or psychosocial factors related to an outcome.
- Published in English language and were either case-control, cross-sectional or cohort study design.

No restriction on publication date was applied. Studies were excluded if they were animal or cadaveric studies, commentaries, editorials, single case studies, reports or laboratory data, books or book chapters, letters, conference posters or proceedings or study protocols. Furthermore, we excluded studies whose participants’ CSp ± R resulted from an upper motor neuron lesion, fracture, radiculitis, myelopathy, post-surgery, whiplash-associated disorder, systemic pathology or metabolic diseases such as diabetes.

### Study identification

We uploaded the search strategy results into the Rayann systematic review online platform (https://www.rayyan.ai). Two reviewers (MM, TS) independently reviewed, checked titles and abstracts and documented decisions on study eligibility. All potentially eligible full-text papers were independently reviewed by the same two reviewers to determine final inclusion. A third reviewer (MT) was available to review any disagreements; this was not required.

### Data extraction

Data extraction forms were designed by the lead reviewer (MM). This form was reviewed and agreed upon by all reviewers. Two reviewers (MM, JT) independently extracted data from included studies. The same two reviewers discussed the data extracted and reached a consensus through discussion. Data extracted included lead author and date of publication; study design; study demographics (country, sample size, age range or mean gender ratio); definition of exposure; report of the comparator; outcome measure description; risk estimates (risk ratios, hazard ratios, odds ratio and mean differences including 95% confidence intervals (CI)) where available.

### Methodological quality

Two reviewers (MM, TS) independently assessed the quality of each included study using a Newcastle-Ottawa Quality Scale (NOS) assessment quality appraisal tool [25]. The NOS checklist assesses the quality of studies across three domains: selection of the studies groups, comparability of the groups and control for confounding factors and exposure. The two reviewers discussed NOS quality appraisal scores and, through discussion, reached a consensus. The certainty of the evidence was assessed as very low, low, moderate or high certainty using the Grading of Recommendations, Assessment, Development and Evaluations (GRADE) [26].

### Synthesis

Two reviewers (MM, TS) assessed all included analyses from a clinical (e.g., diagnosis, variability in population characteristics) and study methodology perspective to determine the suitability of meta-analysis. Both reviewers agreed on the existence of significant clinical heterogeneity, questioning the appropriateness of meta-analysis. Data were, therefore, narratively analysed by patient populations and clinical diagnoses.

## Results

**Fig. 1:**
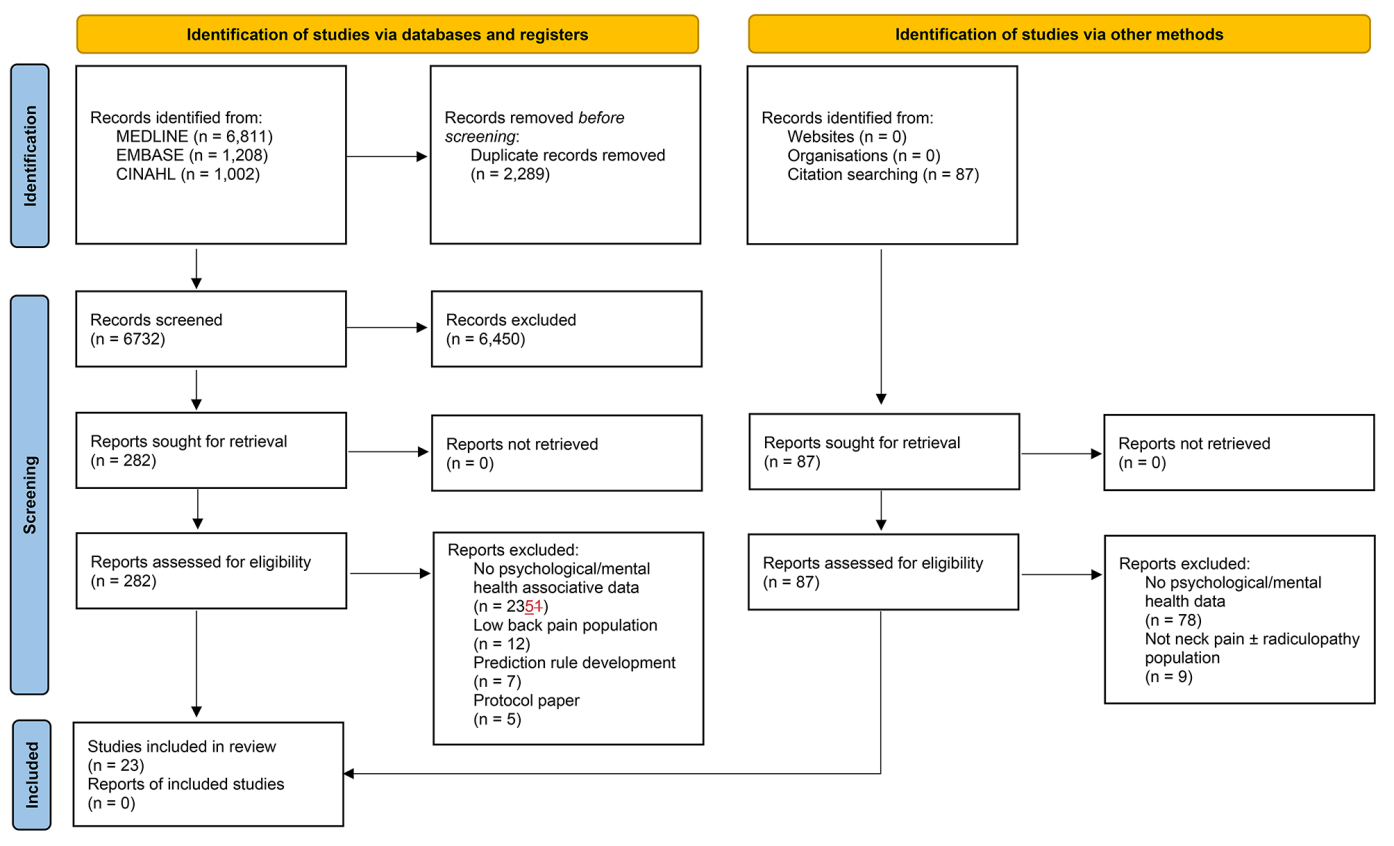
PRISMA 2020 flow diagram for new systematic reviews, which included searches of databases, registers and other sources. *From: Page MJ, McKenzie JE, Bossuyt PM, Boutron I, Hoffmann TC, Mulrow CD, et al. The PRISMA 2020 statement: an updated guideline for reporting systematic reviews. BMJ 2021;372:n71. doi*: 10.1136/bmj.n71

The results of the search strategy are presented in Fig. 1. A total of 6732 studies were identified and screened. Of these 6450 were excluded from the title and abstract. Of the remaining 282 full-text studies reviewed, 259 were excluded. Twenty-three studies met the inclusion criteria and were included in the review [2, 27–48].

### Study characteristics—population and location

A total of 21,968 participants were recruited across the 23 included studies. There were 17,604 participants with non-specific neck pain and 4364 participants with CSR. Sixteen studies included neck pain populations, five were cohort study designs [27, 29, 34, 43, 46] and 11 were cross-sectional in study design [2, 28, 32, 35–37, 39, 41, 42, 45, 47]. Of the seven studies that included CSR populations, five were observational [30, 31, 38, 44, 48] and two were secondary analyses of healthcare records [33, 40]. The characteristics of the included studies are presented in Table 1 (summary study characteristics). A full table of study characteristics can be accessed in Supplementary File 2.

**Table 1:** Summary study characteristics

Author and year	Spinal diagnosis	Mental health diagnosis or symptoms	Health outcome
Alipour (2009)	Non-specific neck pain	Anxiety symptoms regarding changed	Sick Leave from employment
Beltran-Alacreu (2018)	Non-specific neck pain	Kinesiophobia	Presence of pain (NPRS)
Bohman (2019)	Neck pain for 3 months or longer	Depressive symptoms	Neck Disability Index
Carroll (2004)	Non-specific neck pain	Depressive symptoms	Development of pain (NPRS)
Diebo (2018)	Cervical spine radiculopathy	Psychological outcomes with SF-36	Neck Disability Index (NDI)
Divi (2020)	Cervical spine radiculopathy	Psychological outcomes with SF-12	Neck Disability Index (NDI)
Elbinoune (2016)	Neck pain for 3 months or longer	Anxiety and depressive symptoms	Presence of Pain (NPRS)
Engquist (2015)	Cervical radiculopathy	Depressive symptoms	Neck Disability Index
Grimby-Ekman (2012)	Non-specific neck pain	Stress	Presence of pain (NPRS)
Hill (2007)	Non-specific neck pain	Psychological distress	Presence of pain (NPRS)
Hoe (2012)	Non-specific neck pain	Job strain & SF-12 MCS	Presence of pain (NPRS)
Hurwitz (2006)	Non-specific neck pain	SF-36 Mental health	Neck Disability Index
Kim (2018)	Cervical spine radiculopathy	Depressive symptoms	Neck Disability Index and Numeric Pain Rating Score
Lee (2007)	Non-specific neck pain	Psychological distress	Presence of pain (NPRS)
MacDowell (2018)	Cervical radiculopathy	Anxiety and depressive symptoms	Neck Disability Index
McLean (2011)	Neck pain for 3 months or longer	Anxiety and depressive symptoms	Disability of arm and shoulder (DASH)
Meisingset (2018)	Non-specific neck pain	Catastrophising	Pain (NPRS)
Myhre (2013)	Non-specific neck pain	Emotional distress	FABQ-W
Peolsson (2006)	Cervical spine radiculopathy	Distress	Neck Disability Index
Pico-Espinosa (2019)	Non-specific neck pain	Depressive symptoms	Pain levels (NPRS)
Rodriguez-Romero (2016)	Non-specific neck pain	Psychological outcomes with SF-36	Presence of pain (NPRS)
van den Heuvel (2005)	Non-specific neck pain	Job strain	Presence of neck and upper limb pain shoulder pain (NPRS)
Wibault (2014)	Cervical spine radiculopathy	Depression and Anxiety	Neck Disability Index

Seven studies included participants with CSR recruited from elective spinal surgery waiting lists. The CSR diagnosis was made using imaging associated with a neurological deficit on clinical examination [30, 31, 33, 38, 40, 44, 48]. Despite contacting the corresponding authors for further information, no further details were obtained.

Nine studies measured depressive symptoms [2, 29, 32, 33, 38, 40, 43, 45, 48]. Five studies measured anxiety symptoms [27, 32, 40, 43, 48] and three studies measured job-strain and stress [34, 35, 46]. Three studies used the psychological components of SF-36 [30, 39, 47]. Two studies used the psychological components of SF-12 [31, 35]. One study measured kinesiophobia [28] and one study measured catastrophising [41]. Three studies used more than one mental health symptom measurement [32, 35, 43]. A summary of the mental health symptoms and tools to measure the severity of mental health conditions across the 23 included studies are presented in Table 1.

### Neck pain associative outcomes: depressive symptoms

Of the 16 studies with people with non-specific neck pain, there were positive and negative associations between mental health symptoms and health outcomes. Four studies reported a positive association [2, 32, 43], and one study reported a negative association [29] with depression. Using GRADE classifications, the overall strength of evidence was ‘low’, which is attributed to a high risk of bias.

Depressive symptoms measured through Hospital Anxiety and Depression Scale (HADS) was positively associated with the Disabilities of the Arm, Shoulder and Hand (DASH) questionnaire (r:0.245, p = 0.004) [43], Odds Ratio (OR): 3.46 (95% CI: 2.01–5.95) [45] and OR: 1.02 (95% CI: 0.98–1.06) [32]. When measured through the Center for Epidemiologic Studies Depression Scale (CES-D), depressive symptoms were positively associated with pain (Hazard Ratio (HR): 3.97, 95% CI: 1.81–8.72) [2]. Depressive symptoms measured by the Montgomery Asberg Depression Rating Scale were negatively associated with Neck Disability Index (NDI) (OR: 0.94, 95% CI: 0.86–1.03) [29].

### Neck pain associative outcomes: anxiety symptoms

Anxiety symptoms were positively associated with poorer health outcomes in two studies [27, 32] and had no significance in one study [43]. The overall strength of evidence was ‘very low’ in the GRADE assessment which is attributed to a high risk of bias and imprecision.

Anxiety symptoms measured through the Nordic musculoskeletal questionnaire were more likely to be associated with sick leave (OR: 1.4, 95% CI: 0.9–2.1) [27]. Anxiety symptoms measured through HADS were more likely to be associated with the presence of pain (OR: 1.02, 95% CI: 0.98–1.05) [32]. Whereas in one study, anxiety symptoms measured through HADS had no statistical significance with DASH (r: 0.104, p = 0.220) [43].

### Neck pain associative outcomes: Kinesiophobia

Kinesiophobia was associated with poorer health and the presence of pain (r: 0.566, P = < 0.05) in one study [28].

### Neck pain associative outcomes: Catastrophising

Catastrophising, measured by the catastrophising pain scale, was positively associated with pain (OR: 1.03, 95% CI 0.97–1.09) in one study [41].

### Neck pain associative outcomes: stress

Stress was positively associated with the presence of pain (OR: 0.32, 95% CI: 0.25–0.39) in one study [34].

### Neck pain associative outcomes: job strain

A higher job strain was negatively associated with the presence of pain in the neck and shoulder in two studies (Relative Risk (RR): 1.79, 95% CI: 1.19–2.69) [46] and OR: 1.51 (95% CI: 0.88–2.59) [35]. This was rated as ‘low’ in the GRADE assessment, attributed to imprecision across the studies.

### Neck pain associative outcomes: distress

Distress was positively associated with health outcomes in three studies [37, 39, 42] and negatively associated with health outcomes in two studies [36, 47]. The overall strength of evidence using the GRADE approach is ‘very low’, which is attributed to a high risk of bias and imprecision.

Psychological distress measures were positively associated with the presence of pain when measured by SF-36 (r2: 0.12, p < 0.01) [39] and Hopkins Check List-10 (OR: 2.32, 95% CI: 1.20–3.43) [42]. Similarly, this was positively associated with NDI (OR: 1.75, 95% CI 0.83–3.70) [37]. Two studies reported a negative association between distress and the presence of pain (OR: 0.88, 95% CI: 0.62–1.24) [36] and OR: -0.3, 95% CI -0.4-0.1 [47].

### Cervical spine radiculopathy associative outcomes: depressive symptoms

Of the seven studies with CSR populations, there were both positive and negative associations between depressive symptoms and health outcomes. Three studies reported a negative association [33, 40, 44], whereas one study reported a positive association [38]. The overall strength of evidence using the GRADE approach was ‘very low’, this is attributed to a high risk of bias and imprecision.

Depressive symptoms were positively associated with NDI when measured through the Zung Self-Reporting Scale (NDI with depression 42.8 (High) (SD: 19.9) vs. 20.9 (SD: 15.9), p < 0.0001) [38]. Three studies reported negative associations (OR: 0.71, p < 0.001) [44], regression coefficient 0.25 (95% CI: -0.01-0.50) [40] and risk of depression not being significant (p = 0.3) [33].

### Cervical spine radiculopathy associative outcomes: distress

There were two studies that reported a positive association between SF-36 (p < 0.05) [30] and SF-12 (p = 0.04) [31] and NDI. Whereas one study reported distress being negatively associated with NDI (r2 = 0.80, p = 0.0005) [44]. The overall strength of evidence using the GRADE approach was ‘very low’. This is attributed to a high risk of bias and imprecision.

### Cervical spine radiculopathy associative outcomes. Anxiety symptoms

In one study, anxiety symptoms were positively associated with NDI in CSR populations (OR: 0.63, p = 0.006) [48]. All associative outcomes data are populated in Table 2.

**Table 2:** Associative data between health outcome and mental health

Author and year	Associative data between health outcome and mental health
Alipour (2009)	OR: 1.4 (95% CI: 0.9–2.1)
Beltran-Alacreu (2018)	Association kinesiophobia and presence of pain (r = 0.566)
Bohman (2019)	OR: 0.94 (95% CI: 0.86–1.03)
Carroll (2004)	Hazard Rate Ratio 3.97 (95% CI 1.81–8.72)
Diebo (2018)	When NDI is low MHC = 25.81 (SD: 8.85) When NDI is high MCS = 25.60 (SD: 8.87)
Divi (2020)	MHC low score 23.9 (95% CI: 21.0-26.7) vs. MHC high score 31.8 (95% CI: 24.7–38.9) (p = 0.04)
Elbinoune (2016)	HADS-Anxiety OR: 1.02 (95% CI: 0.98–1.05) HADS-Depression OR: 1.02 (95% CI: 0.98 to 1.06)
Engquist (2015)	No risk of depression 4 (95% I: -4 to 15) At risk of depression 10 (95% CI: 1–19) (p = 0.3)
Grimby-Ekman (2012)	OR 0.32 (95% CI: 0.25–0.39)
Hill (2007)	OR 0.88 (95% CI: 0.62–1.24)
Hoe (2012)	High Job Strain OR: 1.51 (95% CI: 0.88–2.59) SF-12 Mental Health Component OR: 0.98 (95% CI: 0.96–0.99)
Hurwitz (2006)	OR 1.75 (95% CI 0.83–3.70)
Kim (2018)	NDI Depression 42.8 (SD: 19.9) vs. Low-depression 20.9 (SD: 15.9) (p < 0.0001) NPRS Depression 5.5 (SD: 2.2) vs. Low depression 3.0 (SD: 2.4) (p < 0.0001)
Lee (2007)	SF-36 MCS and Physical activity (r2: 0.12 p < 0.01)
MacDowell (2018)	Regression Coefficient 0.25 (95% CI: -0.01-0.50)
McLean (2011)	Depression r: 0.245 (p = 0.004) Anxiety r:0.104 (p = 0.220)
Meisingset (2018)	OR: 1.03 (95% CI 0.97–1.09)
Myhre (2013)	OR: 2.32 (95% CI: 1.20–3.43)
Peolsson (2006)	NDI r2 = 0.80 to DRAM (p = 0.0005)
Pico-Espinosa (2019)	OR: 3.46 (95% CI 2.01–5.95)
Rodriguez-Romero (2016)	OR: -0.3 (95% CI: -0.4-0.1)
van den Heuvel (2005)	Low job strain RR: 1.00 (95% CI 0.76–1.92) High job strain RR: 1.79 (95% CI 1.19–2.69)
Wibault (2014)	Depression OR: 0.71 (p = < 0.001) Anxiety OR: 0.63 (p = 0.006)

### Quality assessment. Neck pain populations

Five cohort studies included patients with non-specific neck pain as their exposure [27, 29, 34, 43, 46]. These studies scored between five and seven out of nine on the NOS. All studies met the ‘representativeness of exposed cohort’ and ‘adequate follow-up’. All five studies did not complete the ‘assessment of outcome’ item.

Eleven studies were cross-sectional in study design. Scores ranged from five to seven out of nine on the NOS. All studies met the ‘representativeness of exposed cohort’ and ‘adequate follow-up’. All studies did not meet the ‘assessment of outcome’ item. Three studies completed a secondary analysis of data [36, 37, 45]. These studies scored six to seven out of a possible nine. All studies did not meet the ‘demonstration that outcome of interest was not present at the start of study’ item and ‘assessment of outcome’. The overall strength of evidence measured through GRADE is populated in Table 3. The quality assessment tables are populated in Table 4.

**Table 3:** Certainty of evidence. GRADE approach for health outcomes

Study Design	Study lead author	Number of studies/patients	Risk of bias	Imprecision	Inconsistency	Indirectness	Overall strength of evidence	
**Observational** **Neck pain without CSR**								
Depression	Bohman Caroll Elbinoune McClean Pico-Espinosa	5/1,718	High	Serious	Moderate	No seriousness	Low	
Anxiety	Alipour Elbinoune McClean	1/12,415	High	Serious	High	No seriousness	Very low	
Catastrophising	Meisingset	1/70	High	Serious	High	No seriousness	Very low	
Stress	Grimby-Ekman	1/1200	High	Serious	High	No seriousness	Very low	
Job strain	Van den Heuvel Hoe	2/1898	High	Serious	Moderate	No seriousness	Low	
Distress	Lee Hill Hurwitz	3/802	High	Serious	Moderate	No seriousness	Very Low	
Kinesiophobia	Beltran-Alacreu	1/128	High	Serious	Moderate	No seriousness	Low	
**Observation** **CSR**								
Distress	Diebo Divi Peoplsson	3/639	High	Serious	Moderate	No seriousness	Very Low	
Depression	Kim; Peolsson Enquist MacDowell	4/471	High	Serious	Moderate	No seriousness	Very Low	
Anxiety	Wilbault;	1/254	High	Serious	Moderate	No seriousness	Very Low	

Through this, the certainty of the evidence was either increased (upgraded) or decreased (downgraded) against the following five criteria:

(1) Methodological limitations using the Cochrane Risk of Bias tool (downgraded where there was a high risk of bias for three or more items; upgraded where all items demonstrated a low risk of bias);

(2) Indirectness relating to similarity to clinical practice (downgraded where reviewers felt the study design was not generalisable to UK practice; upgraded where study design was generalisable to UK practice);

(3) Imprecision relating to the number of participants and events (downgraded where outcomes reported less than 300 participants or five events; upgraded where effects reported in excess of 450 participants or 20 events);

(4) Inconsistency in effect estimates across trials for a given analysis (downgraded where the CIs were four-times the magnitude of the effect estimate; upgraded where CIs were two-times the magnitude of the effect estimate)

(5) Likelihood of publication bias (downgraded when reviewers observed asymmetry in funnel plot shape; upgraded when reviewers observed symmetry in funnel plot shape)

**Table 4:** Quality assessment scoring for all included studies

Author and year	Representativeness of the exposed cohort		Selection of the non-exposed cohort		Ascertainment of exposure		Demonstration that outcome of interest was not present at start of study		Comparability of cohorts based on the design or analysis	Assessment of outcome	Was follow-up long enough for outcomes to occur	Adequacy of follow up of cohorts	TOTAL STARS
Alipour (2009)	1		1		0		1		2	0	1	0	7
Beltran-Alacreu (2018)	1		1		0		0		0	0	1	1	5
Bohman (2019)	1		1		0		0		0	0	1	1	5
Diebo (2018)	1		1		1		0		2	0	1	1	7
Divi (2020)	1		1		1		0		2	0	1	1	7
Carroll (2004)	1		1		0		0		2	0	1	1	7
Elbinoune (2016)	1		1		0		0		2	0	1	1	6
Engquist (2015)	1		1		1		0		0	0	1	1	5
Grimby-Ekman (2012)	1		1		0		0		2	0	1	1	7
Hill (2007)	1		1		0		0		2	0	1	0	6
Hoe (2012)	1		1		0		0		2	0	1	1	7
Hurwitz (2006)	1		1		0		0		2	0	1	0	6
Kim (2018)	1		1		0		0		2	0	1	1	6
Lee (2007)	1		1		0		0		0	0	1	1	5
MacDowell (2018)	1		1		1		0		2	0	1	1	7
McLean (2011)	1		1		0		0		1	0	1	1	6
Meisingset (2018)	1		1		0		0		2	0	0	0	5
Myhre (2013)	1		1		0		0		2	0	1	1	7
Peolsson (2006)	1		1		1		0		2	0	1	0	6
Pico-Espinosa (2019)		1		1		0		0	2	0	1	1	7
Rodriguez-Romero (2016)	1		1		0		0		2	0	1	0	6
van den Heuvel (2005)	1		1		0		1		2	0	1	0	7
Wibault (2014)	1		1		0		0		2	0	1	1	6

### Quality assessment. Cervical spine radiculopathy populations

Five cohort studies included patients with CSR as their exposure population [30, 31, 38, 44, 48]. These studies scored between six and seven out of a possible nine on NOS. All studies met the ‘representativeness of exposed cohort’ and ‘adequate follow-up’. All five studies did not complete the ‘assessment of outcome’ item. Two studies with a CSR study population were retrospective secondary data analyses where each study scored five [33] and seven [40], respectively. The overall strength of evidence measured through GRADE is populated in Table 3. The quality assessment tables are populated in Table 4.

## Discussion

This is the first systematic review investigating the association of mental health symptoms and conditions with health outcomes in adults with CSp ± R. Our results indicate that depressive symptoms were associated with poorer health outcomes in seven studies classified as with ‘low quality’, four studies with CSR populations and three studies with non-specific neck pain populations. There was no association with depressive symptoms health outcomes in six studies (four studies with CSR populations and two studies with non-specific neck pain populations) with very low quality. Distress and anxiety symptoms were associated with poorer health outcomes in CSR populations and non-specific neck pain in two studies with ‘very low-level’ quality. Stress and higher job strain was negatively associated with poorer health outcomes measured by the presence of pain in two studies with very low quality sampling non-specific neck pain populations. Stress and higher job strain symptoms were not reported in our included studies that sampled CSR populations.

At the time of conducting this research, there was no universal agreement on CSR diagnosis [3, 49]. Therefore, a pragmatic approach was undertaken, and studies with probable or definite CSR diagnoses were adapted from IASP and North American Spine Society [20, 22, 23] (Supplementary file 1). The diagnostic criteria for CSR varied between each included study. Included studies used a combination of subjectively reported symptoms, clinical assessment testing associated with imaging findings assessed by a physician, and/or sensory and motor electrophysiological testing. In line with our protocol [19], the included patients with CSR would have a ‘definite’ CSR diagnosis. All participants with CSR were on an orthopaedic surgery waiting list, which may question the external validity to alternative healthcare settings such as primary care.

It is acknowledged that a recent international e-Delphi study has been published [50] with an agreement on CSR classification criteria. The 12 physiotherapists who participated in the e-Delphi reached a consensus of radicular pain with arm pain worse than neck pain ***and*** paraesthesia or numbness and/or weakness and/or altered reflex ***and*** MRI confirmed nerve root compression compatible with clinical findings [50]. Future research should now be conducted to test the reliability and determine which tools can be used to assess these criteria [50]. Strengthening these CSR diagnostic criteria should facilitate standardisation of assessment criteria across multiple health care professionals globally and enhance pooling of results and conclusions regarding this disabling condition.

Our results indicate that depressive symptoms were associated with poorer health outcomes in seven studies classified as ‘low quality’. Of these, four studies were with CSR populations and three studies with non-specific neck pain populations. There was no association with depressive symptoms health outcomes in six studies of very-low quality (four studies with CSR populations and two studies with non-specific neck pain populations). The mixed association between depressive symptoms and health outcomes across CSR and non-specific neck pain populations may be attributed to a difference in the assessment tools used to measure depressive symptoms.

Although each assessment tool has appropriate psychometric properties to measure mental health symptoms, the mode of delivery to collect these data may influence responses [64, 65]. For example, previous literature suggests people may rate their health and well-being, more favourable in telephone interviews compared to self-reported paper-based questionnaire [64, 65]. Furthermore, it is not clear whether the included studies assessing CSR and non-specific neck pain populations compared participant’s scores to the general population’s normative values or by using cut-off scores to indicate different levels of clinically relevant distress, anxiety and/or depressive symptoms [66]. These two points may provide some reasoning for the mixed association findings reported between nonspecific neck pain and CSR populations.

Comparing this review’s results to other spinal pain populations may enhance our understanding of health outcomes and inform assessment and management strategies. Depressive symptoms or clinical depression are reported to have worse recovery and greater healthcare utilisation, but not pain or work-related outcomes in people with LBP [51]. However, healthcare utilisation was based on one study and depressive symptoms were based on six highly heterogeneous studies [51]. The differences between our reported findings may be attributed to the inclusion of acute episodes of low back pain (pain lasting less than one month), whereas the CSp ± R populations in this review were all persistent in presentation (lasting more than three months).

The symptoms related to CSR are likely to be underpinnings of neuropathic pain mechanisms compared to non-neuropathic mechanisms associated with nonspecific neck pain [67]. It is known that neuropathic pain is associated with more severe pain, higher workplace absenteeism, distress and higher medical costs [67, 68] compared to non-neuropathic pain which is comparable to our review’s findings. However, it is reported that the expectation of recovery for patients with CSR pending operative management, may reduce the psychological impact on health outcomes [63]. The mixed observations across CSR and non-specific neck pain populations may therefore explained by the complex contributing biopsychosocial factors impacting a health outcomes.

The interactions and mechanisms underpinning mental health symptoms, conditions and health outcomes in musculoskeletal pain populations are highly complex [52–54]. Clinical conditions such as spinal pain with or without radiculopathy will have complex interactions and influences that will be unique to each individual [17]. These factors include genetic [55], pathoanatomical [56] and psychological and lifestyle health factors [17, 57]. The complex interactions will influence pain perceptions, levels of distress and, subsequently, health outcomes [58, 59]. Enhancing our knowledge and understanding of mental health symptoms on health outcomes such as disability, function and pain can guide expectations and management strategies for clinicians and patients with CSp ± R. Healthcare providers should continue to assess mental health symptoms in a holistic assessment framework as part of a robust clinical reasoning process. The identification of patients potentially at risk of long-term disability and worse recovery can enhance patient-centred care pathways and may improve health outcomes [60].

We acknowledge limitations in our review. Included studies were written in the English language or those that could be translated. This may have resulted in a publication bias of our included studies by language. Health outcomes in our target populations can often have multidimensional and complex interactions [61, 62], which may be reflected in the variability of single measurement tools in the included studies. Future research should consider the multidimensional factors and develop core outcome measurements when evaluating health outcomes for this patient population.

## Conclusions

This systematic review has reported variable associations between mental health symptoms and diagnosis with health outcomes in people with CSp ± R. Stress, depressive and anxiety symptoms are associated with poorer health outcomes in patients with CSp ± R. However, this is based on a small number of low-quality studies. The low quality can be attributed to wide-ranging diagnostic criteria and population sampling methods. Further research is indicated to standard diagnosis classification criteria for radiculopathy and developing core outcomes to further our understanding of this debilitating condition.

## Electronic supplementary material

Below is the link to the electronic supplementary material.

Supplementary Material 1

Supplementary Material 2

## Data Availability

The datasets used and/or analysed during the current study are available from the corresponding author upon reasonable request.

## List of abbreviations

CES-D: Center for epidemiologic studies depression scale
CSp ± R: Cervical spine pain with or without radiculopathy
CSR: Cervical spine radiculopathy
DASH: Disability of arm and shoulder
GRADE: Grading of recommendations, assessment, development and evaluations
NDI: Neck disability index
NOS: Newcastle Ottawa scale
OR: Odds ratio
SF-36: Short form 36
SF-12: Short form 12
