# Supplementary material for: The association between psychosocial factors and mental health symptoms in cervical spine pain with or without radiculopathy on health outcomes: a systematic review
Source: BMC Musculoskelet Disord. 2023 Mar 28;24:235. doi: 10.1186/s12891-023-06343-8 (PMC10045438; doi:10.1186/s12891-023-06343-8)
Supplement: Supplementary file 1 — Supplementary Material 1 [file 12891_2023_6343_MOESM1_ESM.docx]

Supplementary file 1. Modified Radiculopathy Diagnostic Criteria

**Definite CSR Diagnosis - *Either (i) or (ii)***

1. Acute denervation with EMG studies or sensory changes in dermatomal distribution

AND

Weakness, atrophy or fasciculation in a myotomal distribution *and* Unilateral diminished deep tendon reflexes

1. Abnormal myelography, CT or MRI correlating with radiculopathy *with* neck pain or combined neck and arm pain

OR

Paraesthesia, hyperaesthesia or dysaesthesia in a nerve root distribution or muscle weakness in a myotomal distribution or atrophy

**Probable CSR Diagnosis *- Either (iii), (iv) or (v)***

1. Neck pain, neck and arm pain, paraesthesia, hyperaesthesia or dysaesthesia in a nerve root distribution or muscle weakness in a myotomal distribution or atrophy

*with*

Sensory changes in dermatomal distribution or muscle weakness in a myotomal distribution or atrophy or fasciculation in a myotomal distribution or unilateral diminished deep tendon reflexes

1. Neck pain, neck and arm pain, paraesthesia, hyperaesthesia or dysaesthesia in a nerve root distribution or muscle weakness in a myotomal distribution or atrophy

*with*

Abnormal myelography, CT or MRI correlating with radiculopathy

1. Neck pain or neck and arm pain *with two* from:

(v-i) Sensory changes in dermatomal distribution

(v-ii) Muscle weakness in a myotomal distribution or atrophy

(v-iii) Fasciculation in a myotomal distribution

(v-iv) Unilateral diminished deep tendon reflexes
