# Supplementary material for: The association between psychosocial factors and mental health symptoms in cervical spine pain with or without radiculopathy on health outcomes: a systematic review
Source: BMC Musculoskelet Disord. 2023 Mar 28;24:235. doi: 10.1186/s12891-023-06343-8 (PMC10045438; doi:10.1186/s12891-023-06343-8)
Supplement: Supplementary file 2 — Supplementary Material 2 [file 12891_2023_6343_MOESM2_ESM.docx]

| Author and year | Study location | Total population number | Male (M): Female (F) | Mean age (years) | Spinal diagnosis | Mental health diagnosis or symptoms | Mental health measurement | Health outcome | Associative data between health outcome and mental health |
| --- | --- | --- | --- | --- | --- | --- | --- | --- | --- |
| Alipour (2009) | Sweden | 12,184 | 11,562M; 581F | 29 | Non-specific neck pain | Anxiety symptoms regarding changed | Nordic Musculoskeletal Questionnaire | Sick Leave from employment | OR: 1.4  (95% CI: 0.9-2.1) |
| Beltran-Alacreu  (2018) | Spain | 128 | 23M; 105F | 42.7  (SD: 12.0) | Non-specific neck pain | Kinesiophobia | Tampa Scale Kinesiophobia | Presence of pain (NPRS) | Association kinesiophobia and presence of pain (r= 0.566) |
| Bohman (2019) | Sweden | 89 | 89F | Median 52  (IQR: 47-59) | Neck pain for 3 months or longer | Depressive symptoms | Montgomery-Asberg Depression Rating Scale | Neck Disability Index | OR: 0.94  (95% CI: 0.86-1.03) |
| Carroll (2004) | Canada | 781 | 400M; 381F | 44.5  (SD: 13.5) | Non-specific neck pain | Depressive symptoms | Center for Epidemiological Studies-Depression | Development of pain (NPRS) | Hazard Rate Ratio 3.97  (95% CI 1.81-8.72) |
| Diebo (2018) | USA | 375 | 121M; 254 F | 65.4  (SD: 10.2) | Cervical spine radiculopathy | Psychological outcomes with SF-36 | SF-36 Mental Health Component (MHC) | Neck Disability Index (NDI) | When NDI is low  MHC = 25.81  (SD: 8.85)  When NDI is high  MCS = 25.60  (SD: 8.87) |
| Divi  (2020) | USA | 264 | 123M; 141F | 53 | Cervical spine radiculopathy | Psychological outcomes with SF-12 | SF-12 Mental Health Component (MHC) | Neck Disability Index (NDI) | MHC low score  23.9  (95% CI: 21.0-26.7)  vs  MHC high score  31.8  (95% CI: 24.7-38.9)  (p=0.04) |
| Elbinoune (2016) | Morocco | 80 | 23M; 67F | 51.8  (SD: 11.8) | Neck pain for 3 months or longer | Anxiety and depressive symptoms | Hospital Anxiety and Depression Scale | Presence of Pain (NPRS) | HADS-Anxiety  OR: 1.02  (95% CI: 0.98-1.05)  HADS-Depression OR: 1.02  (95% CI: 0.98 to 1.06) |
| Engquist (2015) | Sweden | 60 | 30M; 30F | 46  (SD:9) | Cervical radiculopathy | Depressive symptoms | Zung Depression Scale | Neck Disability Index | No risk of depression  4 (95% I: -4 to 15)  At risk of depression  10 (95% CI: 1-19)  (p=0.3) |
| Grimby-Ekman (2012) | Sweden | 1200 | 573M; 627F | 19-25 | Non-specific neck pain | Stress | Mood scale | Presence of pain (NPRS) | OR 0.32  (95% CI: 0.25-0.39) |
| Hill  (2007) | UK | 346 | 127M; 219F | 51  (IQR 23-84) | Non-specific neck pain | Psychological distress | SF-12 MCS | Presence of pain (NPRS) | OR 0.88  (95% CI: 0.62-1.24) |
| Hoe (2012) | Australia | 1111 | 15M; 350F | 41.1  (SD: 11.8) | Non-specific neck pain | Job strain & SF-12 MCS | SF-12 Mental health component | Presence of pain (NPRS) | High Job Strain  OR: 1.51  (95% CI: 0.88-2.59)  SF-12 Mental Health Component OR: 0.98  (95% CI: 0.96-0.99) |
| Hurwitz (2006) | USA | 336 | 105M; 231 F | 45.7  (SD: 12.0) | Non-specific neck pain | SF-36 Mental health | SF-36 Mental health | Neck Disability Index | OR 1.75 (95% CI 0.83-3.70) |
| Kim  (2018) | USA | 57 | 29M; 28F | 60  (SD: 12.1) | Cervical spine radiculopathy | Depressive symptoms | Zung self-rated depression scale | Neck Disability Index and Numeric Pain Rating Score | NDI  Depression 42.8 (SD: 19.9)  vs  Low-depression 20.9 (SD: 15.9) (p<0.0001)  NPRS  Depression 5.5 (SD: 2.2)  vs  Low depression 3.0 (SD: 2.4)  (p<0.0001) |
| Lee (2007) | Hong Kong | 120 | 49M 71F | 40.0  (SD: 10.0) | Non-specific neck pain | Psychological distress | SF-36 Mental Component Scale | Presence of pain (NPRS) | SF-36 MCS and Physical activity (r2: 0.12 p<0.01) |
| MacDowell (2018) | Sweden | 153 | 75M; 78F | 47.0 (SD:6.9) | Cervical radiculopathy | Anxiety and depressive symptoms | Hospital Anxiety Depression Scale | Neck Disability Index | Regression Coefficient  0.25  (95% CI: -0.01-0.50) |
| McLean (2011) | UK | 151 | 61M; 90F | 53.9 (SD 14.4) | Neck pain for 3 months or longer | Anxiety and depressive symptoms | Hospital Anxiety Depression Scale | Disability of arm and shoulder (DASH) | Depression  r: 0.245  (p=0.004)  Anxiety  r:0.104  (p=0.220) |
| Meisingset (2018) | Norway | 70 | 28M; 52F | 43.2  (SD: 12.6) | Non-specific neck pain | Catastrophising | Pain Catastrophising Scale | Pain (NPRS) | OR: 1.03  (95% CI 0.97-1.09) |
| Myhre (2013) | Norway | 373 | 200M; 173F | 40.9  (SD: 9.8) | Non-specific neck pain | Emotional distress | Hopkins Check List-10 | FABQ-W | OR: 2.32  (95% CI: 1.20-3.43) |
| Peolsson (2006) | Sweden | 23 | 15M; 8F | 51  (SD: 11.2) | Cervical spine radiculopathy | Distress | Distress and risk assessment method (DRAM) | Neck Disability Index | NDI r2=0.80  to DRAM  (p=0.0005) |
| Pico-Espinosa (2019) | Sweden | 617 | 191M; 426F | 46 | Non-specific neck pain | Depressive symptoms | Hospital Anxiety Depression Scale | Pain levels (NPRS) | OR: 3.46  (95% CI 2.01-5.95) |
| Rodriguez-Romero (2016) | Spain | 206 | 112M; 94F | 23.5  (SD: 4.1) | Non-specific neck pain | Psychological outcomes with SF-36 | SF-36 | Presence of pain (NPRS) | OR: -0.3  (95% CI: -0.4-0.1) |
| van den Heuvel (2005) | Netherlands | 787 | Not reported | Not reported | Non-specific neck pain | Job strain | Self-reported | Presence of neck and upper limb pain shoulder pain (NPRS) | Low job strain  RR: 1.00 (95% CI 0.76-1.92)  High job strain  RR: 1.79  (95% CI 1.19-2.69) |
| Wibault  (2014) | Sweden | 201 | 105M; 96F | 50  (SD: 8.4) | Cervical spine radiculopathy | Depression and Anxiety | Zung self-rated depression scale and  Somatic Perception Questionnaire | Neck Disability Index | Depression  OR: 0.71  (p=<0.001)  Anxiety  OR: 0.63 (p=0.006) |

Supplementary File 2. Table of included studies characteristics
